# Mast Cell Proteases Tryptase and Chymase Induce Migratory and Morphological Alterations in Bronchial Epithelial Cells

**DOI:** 10.3390/ijms22105250

**Published:** 2021-05-16

**Authors:** Frida Berlin, Sofia Mogren, Julia Tutzauer, Cecilia K. Andersson

**Affiliations:** Department of Experimental Medical Science, Lund University, 221 84 Lund, Sweden; frida.berlin@med.lu.se (F.B.); sofia.mogren@med.lu.se (S.M.); Julia.Tutzauer@med.lu.se (J.T.)

**Keywords:** mast cell, tryptase, chymase, bronchial epithelial cells, morphology, migration, proliferation, holomonitor

## Abstract

Chronic respiratory diseases are often characterized by impaired epithelial function and remodeling. Mast cells (MCs) are known to home into the epithelium in respiratory diseases, but the MC-epithelial interactions remain less understood. Therefore, this study aimed to investigate the effect of MC proteases on bronchial epithelial morphology and function. Bronchial epithelial cells were stimulated with MC tryptase and/or chymase. Morphology and epithelial function were performed using cell tracking analysis and holographic live-cell imaging. Samples were also analyzed for motility-associated gene expression. Immunocytochemistry was performed to compare cytoskeletal arrangement. Stimulated cells showed strong alterations on gene, protein and functional levels in several parameters important for maintaining epithelial function. The most significant increases were found in cell motility, cellular speed and cell elongation compared to non-stimulated cells. Also, cell morphology was significantly altered in chymase treated compared to non-stimulated cells. In the current study, we show that MC proteases can induce cell migration and morphological and proliferative alterations in epithelial cells. Thus, our data imply that MC release of proteases may play a critical role in airway epithelial remodeling and disruption of epithelial function.

## 1. Introduction

Mast cells (MCs) have long been recognized as key cells in different pathological conditions and are mainly acknowledged for their detrimental roles in allergies and asthma [1,2]. Both IgE-mediated and non-IgE-mediated activation of MCs can trigger the release of various immunologically active substances [3]. Firstly, MC activation causes the release of preformed mediators that are stored within the mast cell secretory granules (containing, e.g., histamine, tumor necrosis factor and proteases chymase, tryptase and carboxypeptidase A3), followed by the release of lipid mediators (leukotrienes and prostaglandins) and lastly, de novo production of, e.g., chemokines, cytokines and growth factors [4]. Evidence for their role in pathology is the increased presence and activation in or near structures involved in pathophysiology of the lung, such as smooth muscle, glands and epithelium [5].

Based on their granule content of serine proteases tryptase and chymase, MCs are divided into two major subpopulations in humans: MC_T_ (tryptase^+^) and MC_TC_ (tryptase^+^, chymase^+^) [6]. In the healthy human lung, the MC_T_ is the dominating subtype and is readily found in structures such as bronchial mucosa and alveolar parenchyma [7,8]. In various pathological conditions of the lung, such as severe asthma, chronic obstructive pulmonary disease (COPD), cystic fibrosis (CF) and idiopathic pulmonary fibrosis (IPF) the number of MC_TC_ increase and also home into structures that normally have very few MCs, such as the bronchial epithelium [9,10,11,12,13,14,15,16]. The role of intraepithelial MCs and the phenotypic change from MC_T_ to MC_TC_ in airway pathology is, to this point, unknown.

Another main component in the development of chronic respiratory diseases is an abnormal epithelial airway barrier, which produces excessive amounts of pro-inflammatory mediators in response to pathogens and noxious stimuli, resulting in a cycle of early and permanent lung damage, and ultimately chronic lung disease [17,18,19]. It is established that the airway epithelium undergoes dramatic remodeling and loss of function in various respiratory diseases [20,21,22,23]. The remodeling might initially have a protective function but may lead to loss of function if persisting into a chronic disease. Changes of the airway epithelium in airway disease include transformation towards a more proliferative, less differentiated cell type with loss of cell-cell contact [24].

The in vivo function of MC proteases on various types of epithelium is still under investigation and both detrimental and protective roles have been found [25,26,27,28]. Tryptase has, in vitro, been shown to stimulate epithelial cell proliferation, chemotaxis and differentiation [29]. Chymase, conversely, has in some studies been shown to downregulate skin epithelial cell proliferation [30]. Mechanistically, a number of studies have shown that tryptase can cleave intestinal and corneal epithelial tight junction proteins and degrade hemidesmosomes [31,32]. In addition, several studies have shown that tryptase can induce the release of various pro-inflammatory mediators, such as prostaglandin E2 and IL-8 from epithelial cells, and chymase can additionally stimulate mucin expression in airway epithelial cells [29,33,34]. Overall, these findings suggest that MC proteases also might have profound effects on bronchial epithelial cells. Less is however known regarding the effects of these proteases on bronchial epithelial cell function and morphology, or if these changes are beneficial or detrimental.

Therefore, this study aimed to investigate the effect of MC proteases on bronchial epithelial morphology and function using a novel holographic live cell imaging technique in combination with motility-associated gene arrays and studies of cytoskeletal arrangement. In the current study, we show that MC proteases induce cell migration and morphological and proliferative alterations in epithelial cells. Thus, our data implies that MC release of proteases may play a critical role in airway epithelial remodeling and disruption of epithelial function.

## 2. Results

### 2.1. Mast Cell Proteases Alter Cell Growth and Division Rate in Bronchial Epithelial Cells

**Figure 1:**
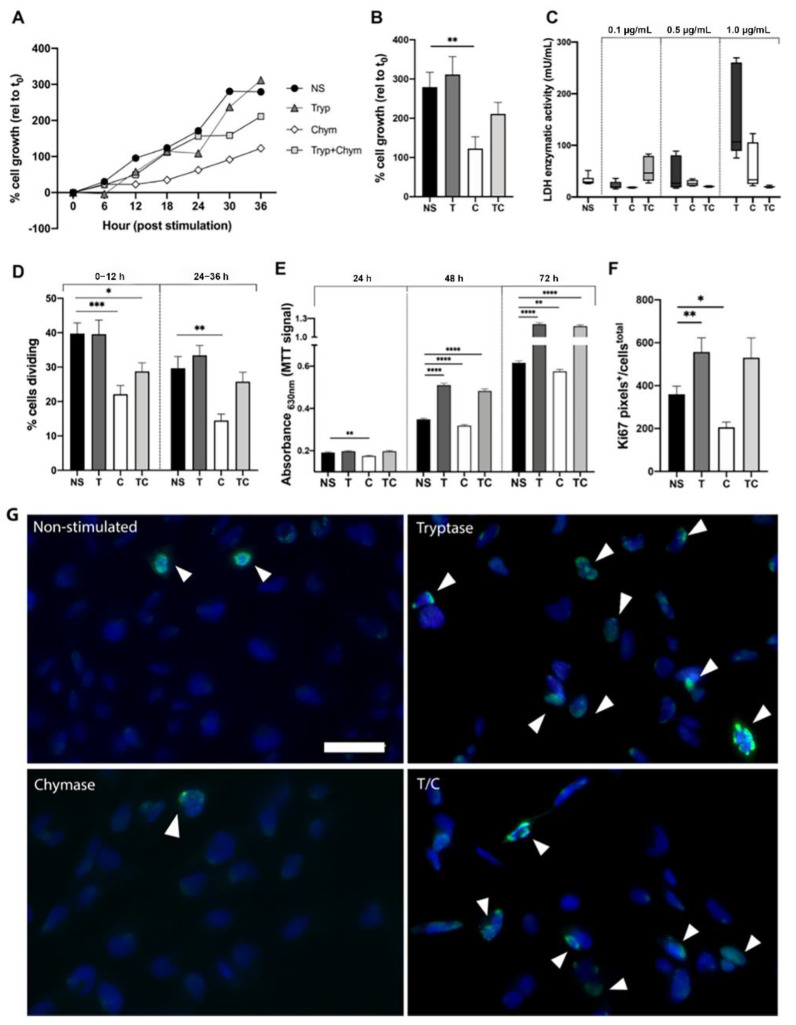
Tryptase induce mitogenic properties whereas chymase attenuate the proliferation rate in bronchial epithelial cells. (**A**) Percentage cell growth over 36 h for non-stimulated cells (NS) or cells treated with tryptase (T), chymase (C) or tryptase and chymase (TC) over time and the percentage of cell increase at 36 h relative to the starting point (**B**) was established using the holographic live cell imaging. LDH enzymatic activity was measured in a dose-response setup (**C**) to detect potential cytotoxicity. (**D**) Percentage of dividing cells at a 12 h interval. (**E**) Representation of MTT signal at 24 h, 48 h and 72 h after treatment. (**F**) Quantification of Ki67 expression (pixels^+^/cells^total^) in BEAS-2B using fluorescence immunocytochemistry and ImageJ. (**G**) Representative micrographs of immunofluorescence stain for KI67 (FITC, green) and nuclei (DAPI, blue) in BEAS-2b stimulated with tryptase, chymase and in combination compared to non-stimulated cells. Scale bar in G: 50 µm. White arrowheads show Ki67 positive cells. Data represent mean ± SEM. * *p* ≤ 0.05, ** *p* ≤ 0.01, *** *p* ≤ 0.001, **** *p* ≤ 0.0001 using Mann–Whitney test. LDH, KI67, MTT: results are based on three independent experiments. Holomonitor: results are based on five focus points per experiment and stimulation, in three independent repeats.

To study the effect of MC proteases tryptase and chymase on epithelial cells, an overall investigation on cell proliferation and cytotoxicity in response to tryptase, chymase or tryptase and chymase in combination was performed. The percentage of cells relative to starting point (t_0_) over time (Figure 1A) and at 36 h (Figure 1B) were obtained using the holographic live cell imaging system. At 36 h, chymase stimulation decreased cell growth (122.5% ± 66.8, *p* = 0.008) and a similar trend was seen for the combination of tryptase and chymase (211.0% ± 65.6, *p* = 0.2). Tryptase induced no significant growth change at 36 h relative to t_0_ (311.6% ± 101.1) in comparison to non-stimulated cells (279.3% ± 84.2, *p* = 0.6). To ensure that the lowered cell growth in chymase treated cells were not due to cell cytotoxicity, a lactate dehydrogenase (LDH) assay was performed, which indicated no cytotoxic effect at the chosen protease concentrations (0.5 µg/mL) (Figure 1C). We also examined images obtained in the Holomonitor to validate the health of the cells and we did not observe any signs of cell death, detachment or floating cells in the wells.

To further investigate the reduced cell growth in chymase-treated cells and the tendency of enhancement of cell growth in tryptase treated cells, the percentage of dividing cells was calculated at 12 h intervals (0–12 h, 24–36 h) of the experiment. In the 0–12 h interval, we found no difference between non-stimulated (40% ± 12) and tryptase treated cells (40% ± 14 *p* = 0.9), but a significant decrease in both chymase (22% ± 8, *p* = 0.0004) and tryptase-chymase treated cells (29% ± 8, *p* = 0.01) in comparison to non-stimulated cells (Figure 1D). At the late time interval, there was still a significant difference between non-stimulated cells (30% ± 11) and chymase (14% ± 6, *p* = 0.001) stimulated cells but not in tryptase-chymase stimulated cells. MTT assay, which measures cell metabolic activity, was performed in order to study cell proliferation (Figure 1E). To assess the long-term effects of MC proteases on cell proliferation, MTT assays were performed at three different time points: 24 h, 48 h and 72 h post-stimulation. Chymase treated cells had significantly decreased in MTT signal after 24 h (0.18 OD ± 0.02, *p* = 0.001) when compared to non-stimulated cells (0.2 ± 0.03). At 48 and 72 h, cells treated with chymase remained significantly decreased (48 h: 0.3 OD ± 0.03, *p* ≤ 0.0001, 72 h: 0.57 OD ± 0.07, *p* = 0.004) whereas tryptase (48 h: 0.5 OD ± 0.05, *p* ≤ 0.0001; 72 h: 1.2 OD ± 0.2, *p* ≤ 0.0001) and tryptase-chymase treated cells (48 h: 0.5 OD ± 0.06, *p* ≤ 0.0001; 72 h: 1.2 OD ± 0.1, *p* ≤ 0.0001) revealed a significant increase in MTT signal in comparison to non-stimulated cells (48 h: 0.35 OD ± 0.03; 72 h: 0.61 OD ± 0.07). Next, quantification of the common proliferation marker KI67 was performed using immunocytochemistry and displayed a significant increase of Ki67 expression in tryptase stimulated cells (557.1 pixels^+^/cells^total^ ± 263.4, *p* = 0.04) and a decrease in chymase treated cells (205.7 pixels^+^/cells^total^ ± 95.0, *p* = 0.01) in comparison to non-stimulated (359.8 pixels^+^/cells^total^ ± 259.9) (Figure 1F,G).

General protease activity and specific tryptase activity were measured at 1, 6, 24, 36 and 72 h after stimulation in cell supernatants to investigate how long the biological activity of the proteases remained throughout the experiment. Using a standard curve setup, both tryptase and chymase showed increasing proteolytic activity with increasing concentrations of chymase and tryptase (Figure S1A,B). General protease activity measurements showed a 48% increase in activity in supernatant from chymase stimulated compared to non-stimulated cells at 1 h. The activity in supernatant from chymase stimulated cells was decreased by 37% at 6 h post-stimulation (Figure S1C). The tryptase activity decreased by 56 and 52% at 6 and 24 h post-stimulation, respectively. At 36–72 h post-stimulation, no tryptase activity was detected in the samples. No tryptase activity was found in non-stimulated cells (Figure S1D).

### 2.2. Bronchial Epithelial Cells Stimulated with MC Proteases Display Cell Elongation and Cytoskeletal Rearrangement

**Figure 2:**
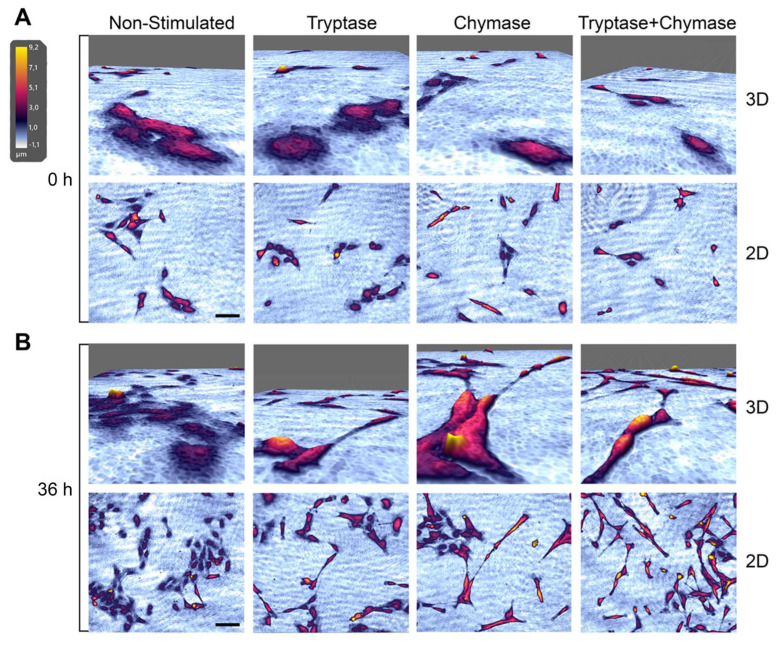
Representative holographic images obtained with HolomonitorM4 at the starting point (**A**) and 36 h (**B)** of five focus points per experiment and stimulation, in three independent repeats. The top images in each panel represent high magnification images in 3D of the bottom 2D images. Scale bar (bottom panels): 100 µm. The colors representing the cell height, where yellow is the maximum cell height.

**Figure 3:**
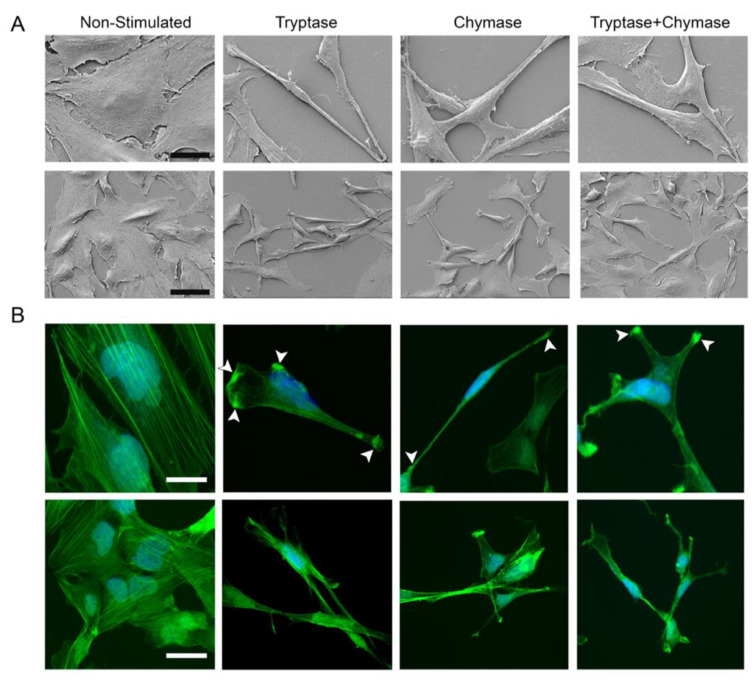
Representative images of epithelial cell morphology in scanning electron microscopy (SEM) (**A**) and confocal microscopy of immunofluorescence cytoskeletal staining using FITC-conjugated phalloidin (**B**). The upper panel in (**A**) and (**B**) represents high magnification images of BEAS-2B. White arrowheads indicate clusters of phalloidin-positive stainings located in the protrusions. Scale bar in A: upper panel 60 µm, lower panel 30 µm and B: upper panel 100 µm, lower panel 50 µm.

The Holomonitor system allowed us to capture 2D as well as 3D reconstructions of cells throughout the experiments. Hence, we compared the cell morphology over time, both within the treatment groups and between the groups. Cells in all treatment groups at 36 h indicated alterations in cell morphology, towards a more elongated shape when compared to non-stimulated cells (Figure 2A,B). To further investigate if tryptase and chymase may induce elongation, cells were treated with tryptase and/or chymase for 24 h and analyzed using a scanning electron microscopy (SEM). High magnification images confirmed the observation from the Holomonitor and demonstrated an increased prevalence of strongly elongated cells in all three treated groups compared to non-stimulated cells (Figure 3A). The non-stimulated cells displayed normal flat, shield-like shapes where cells in close proximity to each other formed a thin but dense monolayer. Cells stimulated with tryptase or chymase, alone or in combination, displayed an elongated, stretched shape in unorganized mono/multilayers. To examine if stimulation with MC proteases was associated with changes in the underlying actin cytoskeleton, we examined the F-actin organization using FITC-conjugated phalloidin and confocal microscopy. A rearrangement of the cytoskeleton was observed in all treated groups, which exhibited an elongated morphology with actin-rich filaments extending into the lamellipodia (Figure 3B, white arrowheads). Non-stimulated cells were less elongated with the actin arranged in multiple long, thin fibers, stretching across the whole cell and exhibited limited lamellipodial structures. The majority of cells stimulated with MC proteases contained numerous lamellipodia projections (T: 70%, C: 65%, TC: 70%), whereas only 3% of non-stimulated cells exhibited this phenotype (Figure 3B).

### 2.3. Tryptase and Chymase Induce Morphological Alterations in Bronchial Epithelial Cells

**Figure 4:**
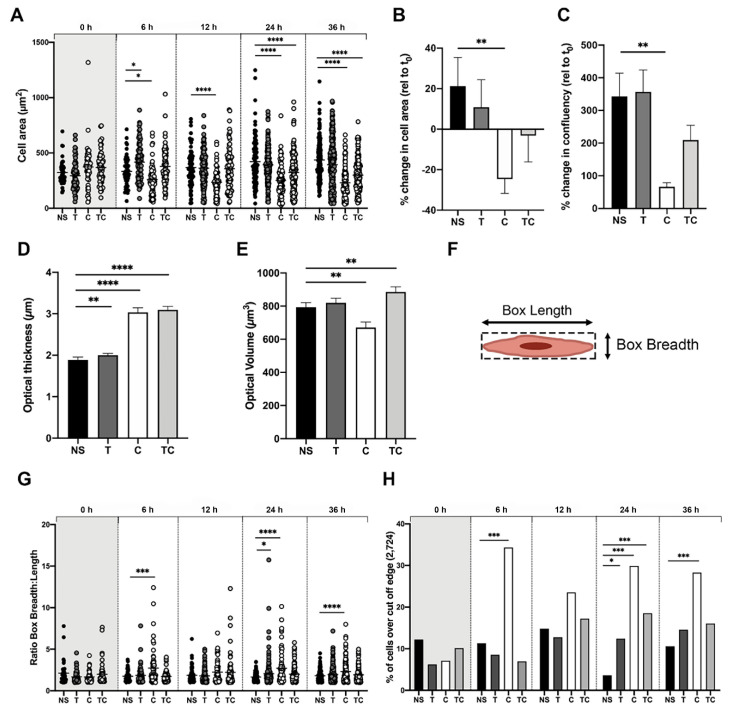
Holographical analysis of morphological alterations in airway epithelial cells stimulated with MC proteases. (**A**) Demonstration of single cell area (µm^2^) over time where each dot represents one cell. (**B**) Cell area at 36 h, relative to starting point. (**C**) Confluency at 36 h relative to the starting point. (**D**) The optical thicknesses and (**E**) optical volume. (**F**) Representation of the measurements of cell box length and box breadth. (**G**) The ratio of box breadth and box length plotted at different time points over time. (**H**) The percentage of cells in each group at different time point that were over the cut-off threshold. The cut-off threshold represents the 10% percentile of the ratio of all NS cells at all time points. Data represent mean ± SEM. Statistical analysis was tested using Mann–Whitney test (* *p* ≤ 0.05, ** *p* ≤ 0.01, *** *p* ≤ 0.001, **** *p* ≤ 0.0001) and for (**G**) Chi-squared test * *p* ≤ 0.05, ** *p* ≤ 0.01, *** *p* ≤ 0.001. Data are based on the same number of cells at each time point and stimulation throughout the different analyzed parameters. Results are based on five focus points per experiment and stimulation in independent repeats.

Using the Holomonitor M4 and its holographic live cell imaging technology, we obtained both 2D and 3D morphological data. When studying cell area over time, we found that the area of chymase treated cells were significantly decreased already after 6 h post-stimulation (260.3 µm^2^ ± 132.7, *p* = 0.04) compared to non-stimulated cells (334.7 µm^2^ ± 133.8) and remained significantly decreased throughout the whole experiment (36 h) (Figure 4A). In contrast, cell area in tryptase treated cells was significantly increased at 6 h (417.5 µm^2^ ± 164.0, *p* = 0.01) compared to non-stimulated cells (Figure 4A). At 24 and 36 h, both chymase and tryptase-chymase treated cells were significantly smaller (24 h; C: 252.0 µm^2^ ± 145.8, *p* ≤ 0.0001; TC: 324.2 µm^2^ ± 173.3, *p* ≤ 0.0001 and 36 h; C: 231.4 µm^2^ ± 135.4, *p* ≤ 0.0001; TC: 299.9 µm^2^ ± 148.1, *p* ≤ 0.0001) in comparison to non-stimulated cells (24 h; 422.8 µm^2^ ± 209.5, 36 h; 435.8 µm^2^ ± 177.4) (Figure 4A). The total change in cell area over time (36 h vs. t_0_) showed a reduction with chymase and a trend towards a reduction with a combination of tryptase-chymase treated cells (C: −24.6% ± 15.6, *p* = 0.03 and TC: −3.2% ± 28.9, *p* = 0.2) (Figure 4B) but no change between non-stimulated cells and tryptase treated cells (NS: 31.2% ± 31.8, T: 10.8% ± 30.4 *p* = 0.5) in relation to their individual starting point. Confluency represents the percentage of the total area of a frame that is covered by cells, and to study the impact of MC proteases, we analyzed the percentage of confluency at 36 h relative to starting time (Figure 4C). We found that chymase-treated cells had a significantly reduced change in confluency (66.8% ± 27.7, *p* = 0.008) at 36 h when compared to non-stimulated cells (NS: 342.4% ± 160.4). No difference was seen for tryptase or chymase and tryptase in combination compared to non-stimulated cells (T: 356.8% ± 149.6, *p* = 0.6; TC: 209.5% ± 100.9, *p* = 0.2). To further investigate 3D morphological alterations, the optical cell thickness at 36 h was analyzed (Figure 4D). We found a significant increased in all three treated groups (T: 2.0 µm ± 0.7, *p* = 0.0002; C: 3.0 µm ± 1.3, *p* ≤ 0.0001; TC: 3.1 µm ± 1.2, *p* ≤ 0.0001) when compared to non-stimulated cells (1.9 µm ± 0.9). When analyzing optical volume, we found no difference between non-stimulated (793.1 µm^3^ ± 371.3) and tryptase (819.6 µm^3^ ± 501.6, *p* = 0.34), but a significant decrease in chymase treated cells (670.3 µm^3^ ± 409.1, *p* = 0.001) as well as a significant increase in tryptase-chymase treated cells (885.4 µm^3^ ± 438.8, *p* = 0.009) (Figure 4E).

To quantify the observations from Figure 2 and Figure 3, cell elongation was evaluated as a ratio between cell box length and cell box breadth (Figure 4E). To evaluate the proteases’ direct effect, as well as effect over time, cell elongation was plotted at different time points (Figure 4F). We found a significant increase in cell elongation already at 6 h in chymase treated cells (2.7 ± 2.1, *p* = 0.003), compared to non-stimulated cells (1.7 ± 0.7). At 24 h after stimulation, both stimulations with tryptase or chymase alone, but not in combination, were significantly increased (T: 2.1 ± 1.5, *p* = 0.04; C: 2.7 ± 1.9, *p* ≤ 0.0001; TC: 2.0 ± 0.9 *p* = 0.53) compared to non-stimulated cells (1.6 ± 0.5). At 36 h, only chymase-treated cells were significantly increased (NS: 1.8 ± 0.7; C: 2.2 ± 1.3, *p* ≤ 0.0001).

Due to the fact that all cells, independent of stimulation, get elongated and round up as they migrate and divide, we also analyzed the top 10% percentile elongated cells at all time points. Here, the top 10% percentile elongation value (2.724) from non-stimulated cells was used as a cut-off threshold, and cells above this limit were considered elongated. The percentage of cells above the threshold in each treatment group and specific time point was calculated ( Figure 4 G). Chymase stimulated cells had a rapid increase in elongated cells and at 6 h post-treatment, stimulation with chymase resulted in 34.3% (*p* ≤ 0.001) elongated cells compared to non-stimulated cells (11.3%). At 24 h, tryptase treated cells (12.4%, *p* = 0.05), chymase treated cells (29.8%, *p* ≤ 0.001) and combination of both (18.5%, *p* ≤ 0.001) were significantly elongated compared to non-stimulated cells (NS: 3.6%). Chymase-treated cells remained significantly elongated even at 36 h compared to non-stimulated cells (C: 28.3%; NS: 10.6%, *p* ≤ 0.001).

### 2.4. Tryptase and Chymase Induce Enhanced Cell Migration, Motility and Speed in Bronchial Epithelial Cells

**Figure 5:**
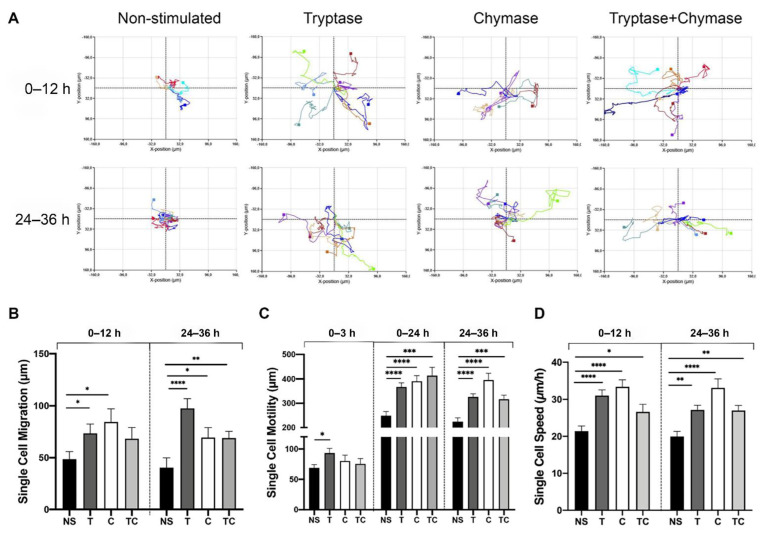
Single-cell tracking showed enhanced cell migration, motility and speed in cells treated with mast cell proteases. (**A**) Representation of single-cell tracking XY-plots obtained from the HolomonitorM4 and compared migration at two different time intervals (0–12 h and 24–36 h) and between stimulation. Individual cells are seen in different colors. (**B**) Comparison of single-cell migration, (**C**) motility and (**D**) speed in tryptase (T), chymase (C) and in combination (TC) compared to controls (NS). Data represent mean ± SEM. * *p* ≤ 0.05, ** *p* ≤ 0.01, *** *p* ≤ 0.001, **** *p* ≤ 0.0001 using Mann-Whitney test. Results based on cell tracking of at least five cells per monitored position and from three randomly chosen focus positions in each well, giving a total of >15 cells per time point and stimulation.

Since both dynamic organization and reorganization of the cytoskeleton and elongated cells are strong indicators of migratory cells, we analyzed MC protease effect on cell motility. We performed single-cell tracking analysis of a minimum of 5 cells per focus point and at three random focus points per stimulation and a total of approximately 5200 analyzed images. Single-cell motility was schemed in an XY plot and illustrated the exact spatial migration of individual cells within one focus point and where each color represents one tracked cell (Figure 5A). We compared the movements obtained between 0–12 h and 24–36 h to monitor both direct effects and changes over time. At the early time point (0–12 h), migration (shortest distance in μm between start point and end point) were significantly increased in tryptase (73.4 µm ± 41.1, *p* = 0.05) and chymase (84.5 µm ± 51.7, *p* = 0.02) treated cells but not with the combination (68.2 µm ± 49.9, *p* = 0.27) when compared to non-stimulated cells (48.56 µm ± 30.2) (Figure 5B). However, at the later time interval, we found a significant difference in all three treated groups (T: 97.6 µm ± 41.6, *p* ≤ 0.0001; C: 69.4 µm ± 40.8, *p* = 0.01; TC: 69.0 µm ± 29.6, *p* = 0.002) in comparison to non-stimulated cells (40.4 µm ± 40.1) (Figure 5B). The single-cell motility analysis (Figure 5C), representing the total distance traveled in μm. Due to an observation of a fast initial in cell motility, we added a time interval (0–3 h) in this analysis. Tryptase treated cells showed a rapid significant onset (0–3 h) of cell motility (93.4 µm ± 34.9, *p* = 0.05) when compared to non-stimulated cells (68.9 µm ± 21.9). No difference was seen for chymase or tryptase and chymase in combination compared to non-stimulated cells (C: 80.2 µm ± 40.7, *p* = 0.9; TC: 75.4 µm ± 40.0, *p* = 0.9). Motility analysis at the later time intervals (0–12, 24–26 h) showed a significantly increase in tryptase (0–12 h: 367.0 µm ± 78.8, *p* ≤ 0.0001; 24–36 h: 326.0 µm ± 57.9, *p* ≤ 0.0001), chymase (0–12 h: 390.5 µm ± 96.7, *p* ≤ 0.0001; 24–36 h: 395.3 µm ± 120.6, *p* ≤ 0.0001) and tryptase-chymase (0–12 h: 413.4 µm ± 158.6, *p* = 0.0004; 24–36 h:316.4 µm ± 80.4, *p* = 0.0004) treated cells when compared to non-stimulated (0–12 h: 249.2 µm ± 69.3; 24–36 h: 224.2 µm ± 67.5). Notably, at the 24–36 h interval, chymase-treated cells migrated approximately double the distance of non-stimulated cells and showed increasingly induced motility over a longer time interval (Figure 5C). Furthermore, in accordance with the motility data, single-cell speed was significantly increased in all three treated groups (Figure 5D), both at 0–12 h (T: 31.0 µm/h ± 7.1, *p* ≤ 0.0001; C: 33.4 µm/h ± 7.7, *p* ≤ 0.0001; TC: 26.6 µm/h ± 9.4, *p* = 0.04) as well as 24–36 h (T: 27.2 µm/h ± 5.4, *p* = 0.01; C: 33.1 µm/h ± 10.2, *p* ≤ 0.0001; TC: 26.9 µm/h ± 6.4, *p* = 0.002) when compared to NS (0–12 h: 21.4 µm/h ± 5.8; 24–36 h: 19.9 µm/h ± 6.0). Taken together, the migratory data obtained from the Holomonitor revealed that chymase induced the highest motility (total travel distance) and migration speed compared to non-stimulated cells at the later time interval. However, tryptase induced the furthest migration directed away from the starting position. The combined stimulation showed results similar to tryptase treated cells except for migration, which was comparable with chymase only treated cells.

An RT^2^ profiler PCR array (PAHS-128Z) for motility-associated genes was analyzed in BEAS-2B after stimulation with tryptase, chymase or in combination (Figure S2 in Supplementary Materials). Tryptase significantly upregulated urokinase plasminogen activator surface receptor (PLAUR, *p* = 0.05) and ezrin (EZR, *p* = 0.03) while downregulated matrix metallopeptidase 9 (MMP9, *p* = 0.006), phospholipase D1 (PLD1, *p* = 0.03) and WASP-family verprolin homologous protein 1 (WASF1, *p* = 0.008). The combination of tryptase and chymase significantly upregulated RAS p21 protein activator 1 (RASA1, *p* = 0.02) and signal transducer and activator of transcription 3 (STAT3, *p* = 0.04). Although not reaching statistical significance, chymase stimulation showed a trend towards upregulating transforming protein RhoA (RHO).

## 3. Discussion

The current study has shown that the MC proteases tryptase and chymase can induce proliferative changes, morphological alterations as well as enhance migration and migration speed in bronchial epithelial cell. Our results are based on gene, protein and functional levels and suggests that MC tryptase and chymase play an important role in airway epithelial remodeling and possible alterations of the epithelial barrier.

MCs are widely acknowledged for their detrimental roles in allergy and asthma [1]. Recent data further show the important roles of MCs in first-line defense against external pathogens [35,36,37]. The detrimental and beneficial impact of MCs are becoming more evident in several pathological settings [26]. In several chronic airway diseases, such as asthma and COPD, the lung MC population is not only increased, but they also undergo a phenotypic switch, from MC_T_ to MC_TC_ [11,12,13]. Intraepithelial MC_TC_s are also increased in these pathological conditions, an occurrence that is rarely seen in healthy subjects or even in mild asthmatics [38,39,40]. Although there are contradictory findings suggesting that chymase contributes to disease pathology, studies have found a positive correlation between MC_TC_ in small airways and bronchial biopsies even in patients with preserved lung function, seen by measurements of FEV_1_/FVC [9,41]. MC_TC_ granules are believed to contain two to three times higher levels of tryptase than granules in MC_T_ [38,42], suggesting that degranulation of MC_TC_s does not only result in the presence of chymase but also a burst of higher concentration of tryptase upon degranulation. An increased MC_TC_ population, also within the epithelium, has been correlated with asthma severity [38]. Various severe respiratory diseases are characterized by epithelial proliferation, remodeling and loss of function. Therefore, we were interested in studying whether chymase, in comparison to tryptase alone or in combination, may play a beneficial or detrimental role in maintaining epithelial homeostasis. To study this, we used the novel holographic live cell imaging system and we found that both tryptase and chymase alone, as well as when stimulated in combination, did have significant effects on the epithelial cell line BEAS-2B.

Previous studies have shown a proliferative effect of tryptase on airway epithelial cells [29]. Chymase has instead been shown to inhibit epithelial cell growth in human skin epithelium [30]. Therefore, we were particularly interested in the proliferative effect on bronchial epithelial cells of tryptase and chymase alone or when the two proteases were combined. Our proliferation data from HolomonitorM4 showed that tryptase alone induced an increase, although not significant, in the number of cells compared to non-stimulated cells during a 36 h experiment. Further, tryptase demonstrated a tendency towards an amplified proliferative result when analyzing division rate at 24–36 h, and a significant increase in proliferation marker KI67 as well as in metabolic activity (MTT), suggesting that tryptase can induce mitogenic properties in bronchial epithelial cells. However, a longer experiment might be required to verify a significantly increased effect at a cellular level. In accordance with previous studies in other epithelial cell types, our data showed a significant decrease in proliferation and cell division rate in chymase-treated cells. Using LDH cytotoxic assay and Holomonitor we found that the decreased cell growth was not due to cell death or cytotoxicity. These effects indicate that chymase might have a detrimental role in epithelial barrier function, leading to diminished healing capacity upon injury. Interestingly, a combination of tryptase and chymase also showed a decreased cell growth curve as well as a decreased percentage of dividing cells at 0–12 h in comparison to non-stimulated cells, but an increase in both KI67 expression and MTT signal, levels that are in line with the observations from tryptase treated cells. This might indicate that chymase has an initial dominant disruptive effect on cell proliferation, which may be reduced or alternatively overtaken by tryptase over time. The release of tryptase and chymase from MCs might play a role in the formation of the metaplastic and hyperproliferative features in bronchial epithelial cells that are observed in biopsies from severe asthmatics [43]. Further investigations of correlations between MC phenotypes and epithelial alterations in airway biopsies from patients with different asthma severity seem warranted.

Typical hallmarks of the airway epithelium in chronic respiratory diseases are increased remodeling and loss of barrier function [26]. Previous studies have shown that intraepithelial MC mediators disturb intestinal epithelial barrier function, remodeling and goblet cell formation [44,45] (reviewed in [46]). Chymase induced dysregulation of the tight junction protein claudin-5, which attenuate intestinal epithelial barrier function [44], but less is known regarding MC specific mediators on airway epithelial cells. In terms of cell morphology, our data shows that both tryptase and chymase alone and when co-stimulated have strong impacts on epithelial cell morphology. Firstly, chymase-treated cells showed reduced cell area over time. Statistically significant effect of tryptase or tryptase-chymase on cell area was not found in comparison to non-stimulated cell. Although a large variety in response should be noted at the later time interval, which might indicate a decline in effect on cell area over time (Figure 1A). To further study cell growth and cell area combined, we analyzed the confluency % relative to t_0_, and in accordance with previous results, chymase reduced cells covering the area. In addition, the optical cell thickness was significantly increased in all three treated groups at 36 h, where chymase induced the strongest response. Given that cell area decreased in combination with an increased cell thickness, the volume of epithelial cells treated with MC proteases was investigated. Indeed, when measuring the optical volume, we demonstrated that chymase-treated cells displayed a substantial decrease in optical volume compared to non-stimulated cells. A significant increase in the volume of tryptase-chymase treated cells compared to non-stimulated cells was found, however. These results suggest that, specifically, MC chymase notably alteration in epithelial cell morphology. Additionally, images from SEM confirm these findings, which shows that cells treated with MC proteases lose their normal shield-like appearance, indicating an alteration of the epithelial barrier and possible effects on the role as the first line of defense. Analysis of the functional consequences of these morphological alterations remains to be investigated. Furthermore, studies of the morphological and functional effects of carboxypeptidase A3 remain an important future research area.

Cells treated with MC proteases displayed a higher number of cells that had an elongated shape (ratio between cell length and breadth). This response was seen early (6 h) in chymase-treated cells, whereas an increase in both tryptase and chymase-treated groups was observed at 24 h post-stimulation. Since all cells, independent of stimulation, elongate over time due to biological cycles and naturally occurring migration when cells are not 100% confluent, the top 10 percentile based on non-stimulated cell elongation was used. Using this approach, more than 30% of all cells treated with chymase were considered elongated. To further evaluate the elongation, we manually assessed epithelial cell elongation using SEM and immunofluorescence microscopy. We found that the proportion of cells as well as the actual cell elongation ratio to be even more increased in all three treated groups when compared to the analyses obtained from the Holomonitor. Furthermore, using SEM, protease-treated cells displayed an unorganized single and multilayer of spindle-like cells with long protrusions. Another observation was that the more elongated a cell was, the less contact it had with other neighboring cells (observational data). Taken together, our results indicate that particularly chymase affects the epithelial cells by reducing epithelial cell growth, single-cell area and shift in cell morphology towards an elongated shape. How this affects the expression of integrins and barrier proteins such as E-cadherin, claudins and zonula occludins remains to be further investigated, both in *in vitro* models and in tissue from patients with increased numbers of epithelial MCs. A disturbed epithelial barrier is likely to trigger immunological inflammation, which further increases in lung tissue permeability and promotes inflammation, which is seen in chronic respiratory diseases.

Tryptase has been reported to enhance cell migration in several different cell types, such as fibroblasts, retinal epithelial cells and eosinophils [34,47,48,49]. To date, little is known regarding chymase pro-migratory effect on human cells in general and in airway epithelial cells in particular. However, two in vitro studies have reported that chymase had no effect on human fibroblasts or inhibitory effects on migration in corneal epithelial cells [48,50]. Further, an *in vivo* study in mice demonstrates a positive correlation between chymase and homeostatic intestinal migration [44]. Although no previous reports have described a direct pro-migratory effect induced by chymase, more light has been shed on its proteolytic activity. Studies have shown that chymase cleaves several proteins central for tissue remodeling and degradation of extracellular matrix (ECM). It acts through direct cleavage of ECM substrates, including fibronectin and procollagen as well as by activating ECM-degrading proteases, such as MMP-2 and -9 [44,51,52,53,54]. Degradation of ECM and tight junctions [31,32] facilitates cell migration and, therefore, chymase might promote cell migration through these mechanisms.

In the current study, we studied the effect of tryptase and chymase alone as well as the simultaneous effect of the two proteases on epithelial cell migration. In contrast to the few previous studies, we found a strong enhancement of cell migration, motility and speed when stimulating cells with either tryptase, chymase or the combination of the two proteases. By using the holographic live cell imaging system, we were able to carefully track individual cells at an interval of 15 min over 36 h (representing >5000 images in total). Hence, this method gave us a very precise illustration of cell movements, both visually through videos as well as in automated measurements of exact values which were used for analysis. Interestingly, we found that tryptase-stimulated cells had an earlier onset of enhanced cell motility. Already at 3 h post stimulation, the distance was significantly increased in comparison to non-stimulated cells, suggesting a rapid and direct migratory effect mediated by tryptase. However, over a 12 h interval (0–12 h) the chymase-treated cells also showed significantly increased migration. Since the migration, motility and speed were still significantly increased at the later time interval (24–36 h post stimulation), our data suggest that MC protease stimulation does not only induce a rapid burst of cell movement, but also a relatively persistent effect. Additionally, chymase induced a consistently high migratory phenotype over all time intervals, whereas non-stimulated, tryptase and tryptase-chymase treated cells slightly lost this phenotype over time. This implies that the two MC proteases do not act pro-migratory through identical signaling pathways, however, the mechanism remains to be investigated. Moreover, seen from a clinical perspective, the remarkable enhancement of epithelial cell migration induced by both tryptase and chymase that was shown in this study, implies a crucial role of intraepithelial MCs as well as a consequence of the shift from MC_T_ to MC_TC_ on epithelial remodeling and homeostasis. However, in an *in vivo* setting the proteases might be degraded at a higher speed than in an *in vitro* setting. Noteworthy, the pro-migratory effect induced by chymase might be limited to specific cell types, such as epithelial cells, since chymase did not have any pro-migratory effects in fibroblasts [34,49] but a clear increase in cell migration in BEAS-2B.

An RT^2^ profiler PCR array for motility-associated genes showed that tryptase and chymase significantly upregulated several important genes inducing migration and cytoskeletal alterations in cells. In our study, the gene expression for the receptor for urokinase plasminogen activator receptor, PLAUR, was upregulated by tryptase. PLAUR has been identified as an asthma-associated gene showing association between single nucleotide polymorphisms and asthma susceptibility, bronchial hyper-responsiveness (BHR) and decline in lung function [55]. Furthermore, patients with asthma had increased tissue expression of epithelial PLAUR [56]. Altered expression has been shown to lead to distorted wound repair, which may contribute to airway remodeling in asthma. Another tryptase upregulated gene identified in this study is ezrin. Results have shown that ezrin can contribute to asthma by affecting bronchial epithelium repair via RhoA pathways [57]. In the present study, there was a trend towards upregulation of RhoA in BEAS-2b when stimulated with chymase. RhoA is a main regulator of cell shape changes in epithelial cells and controls actin assembly and contractility [58]. It is well known that RhoA/Rho-kinases play an important role in the pathophysiology of asthma, including airway hyper-responsiveness and airway remodeling [59]. Interestingly, RhoA also have effects on irreversible junction deformations, which lead to adhesion receptor remodeling possibly affecting epithelial barrier properties [60,61]. Taken together, our gene array data showed that MC proteases chymase and tryptase have broad effects on basic epithelial functions such as cell-cell contact and repair and support our functional data. Since the gene data are based on relatively few repeats, which limits the detection of significant results, in-depth analyzes of the genes identified by this array needs to be further investigated.

Protease activity was measured to investigate how long the biological activity remained in the cell supernatants throughout the duration of the experiment. The results indicate that both tryptase and chymase used in the study were active proteases, as seen in the standard curve measurements for respective protease. Our data showed that at 6 h post-stimulation, the activity of both proteases was reduced by approximately half compared to 1 h post-stimulation. At 36–72 h post-stimulation, no tryptase activity remained in the samples. This may indicate that the cellular changes observed in BEAS-2b induced by tryptase and chymase are initially dependent on the biological activity of the proteases. The specific activity of chymase is difficult to measure since many substrates are unspecific and are cleaved by all chymotrypsin-like proteases. However, since exogenic proteases were added and a negative control was included (non-stimulated cells), we measured the relative activity at 1, 6, 24, 36 h and 72 h post-stimulation using measurements of general protease activity. To further increase the relevance of the chymase activity measurement, we included a standard curve based on chymase activity in our assay.

We have for the first time studied the role of MC tryptase and chymase on airway epithelial cell morphology and function by using the novel holographic live cell imaging system, Holomonitor M4. This method is label-free and is performed inside an incubator, allowing minimal interference with the cells. Some exclusive strengths of using this system are the ability to study 3D reconstructed cells over a certain time lap, obtain multiple morphological and quantitative cell parameters at each captured frame, and to get a great overall picture of cell behavior and health. Additionally, the cell tracking system enables a unique possibility to analyze the accurate cell migration, motility and speed over time. Furthermore, we complemented our observations with high magnification imaging using SEM and immunofluorescence microscopy to allow a detailed demonstration of cell morphology. One limitation of the study is the use of the bronchial epithelial cell line BEAS-2B. Although the BEAS-2b cell line is used extensively as a substitute for airway epithelial cells, this cell line is derived by transforming human bronchial epithelial cells with an adenovirus 12-simian virus 40 construct [62]. In the process of immortalization, some airway epithelium characteristics might have been lost. In addition, BEAS-2b cells were grown in submerged culture, another difference from primary cells in air–liquid interface cultures that may contribute to an altered response in the less differentiated BEAS-2b. However, BEAS-2b has been widely used in various in vitro cell models in a large variety of studies associated with respiratory diseases, including asthma, COPD and lung carcinogenesis, with a relevant pro-inflammatory response to various stimuli. The usage of an epithelial cell line was important in this study due to being stable, with little biological variation between experiments. Since this is the first time MC protease–epithelial cell interactions have been studied in a live-cell imaging setup, and thus, of importance. However, studies of primary cell cultures will be an important future continuation of this study.

In this study, we have used a concentration of 0.5 ug/mL of tryptase and chymase, and this dose was chosen with regard to being non-toxic but still giving robust alterations in cell function and morphology. Further, it gives a clear pro-inflammatory response (Ramu et al., BMC Immunology, 2021) in BEAS-2b. It is, however, difficult to determine how much tryptase is released in vivo, as well as the local concentration around each epithelial cell when MCs degranulate. MCs can secrete mediators in various ways, for example, through piecemeal or anaphylactic degranulation [63], and the effects on the airway epithelial cells are likely to vary with protease concentration. Due to that MC proteases are secreted in large aggregates, complexed with serglycin proteoglycans, and these complexes tend to accumulate locally [26], the local concentration of MC mediators is likely to be quite high. MCs are also present in healthy human lung tissue and, therefore, it is of interest to also investigate what effect a subtoxic dose of MC proteases have on epithelial cells [7]. Furthermore, with regard to the combined dose of both tryptase and chymase, we cannot exclude that any functional changes on epithelial cells are due to the biological activity of the enzymes interacting with each other. However, as MC_TC_ are likely to release both proteases when activated, it is still relevant to study them in combination.

In conclusion, we show that MC proteases, and in particular chymase, can induce cell migration and morphological alterations in bronchial epithelial cells. Given the low presence of MCs, and especially chymase-positive MCs within or near the epithelium in healthy individuals, this might have strong implications on chronic respiratory diseases where these cells increase in numbers. As far as we are aware, this is the first study to investigate tryptase, chymase and the interactive effect of the two MC proteases on airway epithelial cells using holographic live-cell imaging. Thus, our data implies that intraepithelial MC release of proteases may play a critical role in airway epithelial remodeling and pathological alterations of epithelial function.

## 4. Materials and Methods

### 4.1. Cell Culture

Human bronchial epithelial cells, BEAS-2B (ATCC, Walkersville, MD, USA) were maintained in RPMI-medium 1640 (Gibco, Paisley, UK, 61870-010) with a supplement of 10% fetal bovine serum (FBS) and 1% penicillin–streptomycin (Life Technologies, Stockholm, Sweden). The cells were cultured in a humidified atmosphere at 37 °C in 5% CO_2_ until approximately 80–90% confluency was reached. MC proteases were diluted in starvation medium containing RPMI medium with 1% FBS and 1% penicillin–streptomycin. A harvest of supernatant, RNA and protein cells were collected after 6 and 24 h post-stimulation. For Holomonitor experiments, cells were treated with tryptase and/or chymase diluted in starvation RPMI-medium and thereafter monitored for 36 h.

Tryptase: Cells were stimulated with 0.5 µg/mL tryptase (Merck Millipore, Darmstadt, Germany). A highly purified tryptase was used, with a molecular weight of ~135 kDa and a non-covalently linked tetramer with two sets of dissimilar subunits of a and b isoforms sequences. The monomer molecular weight was 31–33 kDa. The undiluted tryptase has an activity of 62.5 units/mg protein (unit definition: one unit will hydrolyze 1.0 μmole of N-benzyl-DL-Arg-pNA per minute at pH 8 at 25 °C).

Chymase: Cells were stimulated 0.5 µg/mL recombinant human chymase (Sigma-Aldrich, Saint Louis, MO, USA). The undiluted chymase had an activity of 90 units/mg protein (unit definition: one unit hydrolyzes one micromole of N-benzoyl-L-tyrosine ethyl ester (BTEE) per minute at pH 7.8 and 25 °C).

Co-stimulations: cells were stimulated using 0.5 µg/mL tryptase and 0.5 µg/mL chymase.

### 4.2. Tryptase and Chymase Activity Measurements

For the tryptase activity measurements, a mast cell degranulation assay kit (IMM001, Merck Millipore) that is based on the spectrophotometric detection of the chromophore p-nitroaniline after cleavage of the substrate tosyl-gly-pro-lys-pNA was used. A positive control for tryptase was included in the experimental setup. A general protease activity kit (EnzCheck Protease assay kit, BODIPY FL E6638, Molecular Probes/Invitrogen) was used to measure chymase activity. To further increase the relevance of the chymase activity measurement, a standard curve based on chymase activity to our assay was included. In all measurements, a negative control (non-stimulated cells) was used as a baseline and activity was interpreted relative to activity in non-stimulated cell supernatant.

### 4.3. LDH Assay

The cytotoxicity was measured in cell supernatants (collected 24 h post-stimulation) using a lactate dehydrogenase (LDH) assay (Roche Diagnostics, Basal, Switzerland). The assay was performed according to manufacturer’s protocol. Briefly, the LDH activity is direct proportional to the enzymatic reaction which can be measured in absorbance.

### 4.4. RNA Extraction and RT2 PCR Array

BEAS-2B were cultured in 12 well Nunc multidish (Nunc Technologies, Carlsbad, CA, USA) and total RNA was collected 6 h post MC protease stimulation. RNA was isolated using RNeasy Plus Micro Kit from QIAGEN (Hilden, Germany), followed by RNA measurement with nanodrop 2000c Thermo Scientific (Waltham, MA, USA). Thereafter mRNA was reversed transcribed to cDNA (RT2 First Strand Kit, QIAGEN, Hilden, Germany) and real-time quantitative PCR was carried out (Agilent Technologies Stratagene MX 3005P) using QIAGEN RT^2^ profiler PCR array (PAHS-128Z) for genes associated with human motility pathways.

### 4.5. MTT Assay

Proliferation rate was evaluated using the 3-(4,5-dimethylthiazol-2-yl)-2,5-diphenyl tetrazolium bromide (MTT) assay. Cells were seeded at a density of 15,000 cells/well to a transparent, flat-bottomed 96-well plate. After adherence overnight, cells were treated with tryptase and/or chymase for 24, 48, or 72 h. On the day of analysis, 10 *v*/*v*% of MTT (5 mg/mL, Sigma, #M2128) was added to each well and incubated for 4 h at 37 °C. The medium was gently aspirated using a hypodermic needle and formazan crystals were dissolved in 100 µL of isopropanol, 0.04 N HCl, and homogenized on a shaker for 10 min. MTT absorbance was measured using a CLARIOstar microplate reader (BMG LabTech, Ortenberg, Germany) at 630 nm, with a background reading at 590 nm. After subtraction of the background, the values were plotted.

### 4.6. Immunocytochemistry

For fluorescence visualization of the nuclei epithelial expression of KI67 or phalloidin, BEAS-2B were cultured in 4 well chamber slides (Merck Millipore, Darmstadt, Germany). When confluency around 60% was reached, the cells were treated with tryptase and/or chymase for 24 h followed by 20 min fixation with 2% paraformaldehyde and rinsing with PBS. In order to enable antibody access to intracellular target protein the samples were permeabilized in PBS containing 0.1% Tween-20. Samples were immunostained with antibodies for proliferation target KI67, dilution 1:300 (Dako, Glostrup, Denmark) and thereafter labelled with Alexa Fluor 488, dilution 1:200 (Invitrogen, Eugene, OR, USA). Isotype (Dako) and negative control (omitting the primary antibody) was used for validation of the antibody and did not give any staining. For cytoskeletal stainings, samples were immunostained 1:1000 with FITC labeled phalloidin 488 (Sigma-Aldrich, St. Louis, MO, USA). Nuclei counterstaining was performed using mounting medium ProLong Gold antifade reagent with DAPI (Invitrogen, Eugene, OR, USA). For KI67 quantification, pictures were captured using Nikon Eclipse 80i (Melville, NY, USA) combined with Nikon DS-QI1MC, and image analysis was carried out using ImageJ (Madison, WI, USA) by measuring the pixel intensity in relation to the threshold value, which was determined based on non-stimulated controls. The pixel intensity value in five randomly-chosen 10× magnification areas and then divided by the number of cells (based on nuclei DAPI staining) in each field of view per repeat and stimulation. For visualization of cytoskeletal organization images were collected using a Nikon Eclipse TE2000-E confocal fluorescence microscope (Nikon, Tokyo, Japan) with an immersion oil × 0.75 numerical aperture objective (Nikon) and pinhole large. The number of cells positive for lamellipodia was counted in five randomly chosen areas and then divided by the number of total cells (based on nuclei DAPI staining) in each field of view. Slides were decoded and three different researchers performed the analysis independent of each other. Images were analyzed with ImageJ software.

### 4.7. Scanning Electron Microscopy (SEM)

The samples were viewed with scanning electron microscopy and prepared according to local standards and protocol. Briefly, BEAS-2B was fixed in approximately 10 times the sample volume of fixation solution containing 0.1M Sorensen’s phosphate buffer pH 7.4, 1.5% formaldehyde and 1.5% glutaraldehyde at room temperature for 20 min. After fixation, the samples were washed twice in 0.1M Sorensen´s buffer pH 7.4 to remove excess fixative. Samples were then dehydrated in a graded series of ethanol (50%, 70%, 80%, 90% and twice in 100%) and subsequently, critical point dried before being mounted and examined in a Jeol JSM-7800F FEG-SEM.

### 4.8. Live Cell Imaging: Migration, Morphology and Proliferation

Functional studies of epithelial migration, morphology and proliferation was per-formed with Holomonitor M4 live cell imaging system from Phase Holographic Imaging (Lund, Sweden). BEAS-2B were cultured in Sarstedt TC 6-well plate (Nümbrecht, Germany) and the imaging was performed inside an incubator at 37 °C in 5% CO_2_. Prior to the start of monitoring, a capture pattern of 5 randomly chosen positions per well and time lapse for imaging (one image every 15 min over 36 h) for respective wells was selected. One 6-well plate resulted in approximately 5200 images. The quantification of the automatic time lapse and cell tracking to determine migration, morphology and proliferation in multiple independent repeats and was carried out using HStudio, a software system with a capacity of a wide range of applications designed for the analysis of holographic microscopy images of unstained adherent cells, using Holomonitor. For the automated cell recognition, the same settings and thresholds were used for treatment groups and non-stimulated cells.

In the present study following parameters were evaluated:

○ Cell growth over time (%): The cell count was based on the automatic cell identification in the HStudio and describes the number of cells per captured image. This represents the percentage cell growth relative to the starting point, which was calculated by dividing the cell count at a certain time point with the starting cell count for that specific focus point. The chosen time points were 0, 6, 12, 24 and 36 h.
○ Confluency (%): The confluence was based on the cell area identification and describes the total area of a frame that is covered by cells. The confluency at 36 h was divided by the confluency at t_0_ and represents the increase of cell confluency at 36 h relative to starting value.
○ Dividing cells: The percentage of dividing cells was obtained by calculating the number of dividing cells within the time interval divided by the starting number of cells at the beginning of the time interval.
○ Cell Area (µm^2^): cell area was obtained using cell area identification application in HStudio and was based on calibration and calculation of single pixels. The single-cell area at different time points over 36 h as well as the relative change in cell area at 36 h relative to the starting value was plotted.
○ Cell elongation: Cell elongation is represented as a ratio between box length and box breadth. It was calculated by dividing the cell box length with box breadth for each cell and then individually plotted at different time points over 36 h. The 10% percentile was calculated by collecting all cell ratios of the non-stimulated cell across all time points and analyzed in GraphPad Prism version 9.0 (GraphPad Software, La Jolla, CA, USA). The obtained value was then used as cut-off threshold, and the percentage of cells above and below this threshold was calculated and plotted.
○ Optical volume (µm^3^): represents the single cell volume and is calculated from the phase shift and is independent of cell shape.
○ Optical thickness (µm): represents the maximum cell thickness and is obtained from maximum phase shift.
○ Cell movement: Cell movement is categorized both by non-directional cell motility and by directional cell migration.
  ▪ Migration (µm): measurement of the linear distance that a cell moves between starting position A and end position B of the cell path. The parameter indicates the directness of cell movement.
  ▪ Motility (µm): measurement of the total distance traveled per individual cell. This measurement will quantify the full path of the movement of the cell. However, it will not measure how far from the starting position the cell has moved, but rather the overall activity of movement. Cell motility is regarded as random cell movement, occurring in almost every cell culture.
  ▪ Cell speed (µm/h): measurement of how far the cell moves in µm per time unit (hour). Data was based on the movement of a cell from one analysis frame to the next and divided by the time between the frames (15 min).

### 4.9. Statistical Analysis

Data were analyzed with GraphPad Prism version 9.0 (GraphPad Software, La Jolla, CA, USA). To determine differences between non-stimulated and tryptase, chymase and in combination, respectively, nonparametric Mann–Whitney U test was used. Chi-squared test was used for analyzing cell elongation. Results were considered significant at *p* ≤ 0.05.

## 5. Conclusions

Our data implies that MC proteases can enhance cell migration and induce alterations in cell proliferation and cell morphology. Hence, our results suggest that intraepithelial MC release of tryptase and chymase might have important roles in airway remodeling and disturbing epithelial function. Further analysis is now warranted on these effects in primary cells and tissue from patients with chronic respiratory diseases.

## Data Availability

The datasets during and/or analyzed during the current study are available from the corresponding author on reasonable request.

## Supplementary Materials

The following are available online at https://www.mdpi.com/article/10.3390/ijms22105250/s1, Figure S1: General protease activity and specific tryptase activity measured at 1, 6, 24, 36 and 72 h after stimulation in cell supernatants, Figure S2: Tryptase and chymase induce alteration in motility associated genes in bronchial epithelial cells.

## Abbreviations

C	chymase
CF	cystic fibrosis
COPD	chronic obstructive pulmonary disease
ECM	extracellular matrix
EZR	ezrin
IPF	idiopathic pulmonary fibrosis
LDH	lactate dehydrogenase
MC	mast cell
MMP	matrix metalloproteinase
MTT	3-(4,5-dimethylthiazol-2-yl)-2,5-diphenyl tetrazolium bromide
NS	non-stimulated
OD	optical density
PLAUR	urokinase plasminogen activator surface receptor
PLD1	phospholipase D1
RASA1	RAS p21 protein activator 1
RHO	transforming protein RhoA
SEM	scanning electron microscopy
STAT3	signal transducer and activator of transcription 3
T	tryptase
TC	tryptase-chymase
WASF1	WASP-family verprolin homologous protein 1
