# Supplementary material for: Mast Cell Proteases Tryptase and Chymase Induce Migratory and Morphological Alterations in Bronchial Epithelial Cells
Source: Int J Mol Sci. 2021 May 16;22(10):5250. doi: 10.3390/ijms22105250 (PMC8156481; doi:10.3390/ijms22105250)
Supplement: Supplementary file 1 [file ijms-22-05250-s001.zip › ijms-1184373-supplementary.pdf]

Online supplement

Mast Cell Proteases Tryptase and Chymase induce Migratory and Morphological Alterations in Bronchial Epithelial Cells

Frida Berlin, Sofia Mogren, Julia Tutzauer and Cecilia K Andersson *

Department of Experimental Medical Science, Lund University, 221 84 Lund Sweden; frida.berlin@med.lu.se (F.B.); sofia.mogren@med.lu.se (S.M.); Julia.Tutzauer@med.lu.se (J.T.); cecilia.andersson@med.lu.se (C.K.A.)

* Correspondence: cecilia.andersson@med.lu.se; +46-46-222-77-46

Results

1.1 Measurements of general protease and tryptase activity in cell supernatants from BEAS-2b after stimulation.

General protease activity and specific tryptase activity was measured at 1, 6, 24, 36 and 72 h after stimulation in cell supernatants to investigate how long the biological activity of the proteases existed throughout the experiment. Using a standard curve setup, both tryptase and chymase showed increasing proteolytic activity with increasing concentrations of chymase and tryptase (Figure S1A-B). General protease activity measurements showed an 48% increase in activity in chymase stimulated compared to non-stimulated cells at 1 h. This activity was decreased by 37% at 6 h post stimulation (Figure S1C). The tryptase activity decreased by 56 and 52% at 6 and 24 h post stimulation, respectively. At 36-72 h post stimulation, no tryptase activity was detected in the samples. No tryptase activity was found in non-stimulated cells (Figure S1D).

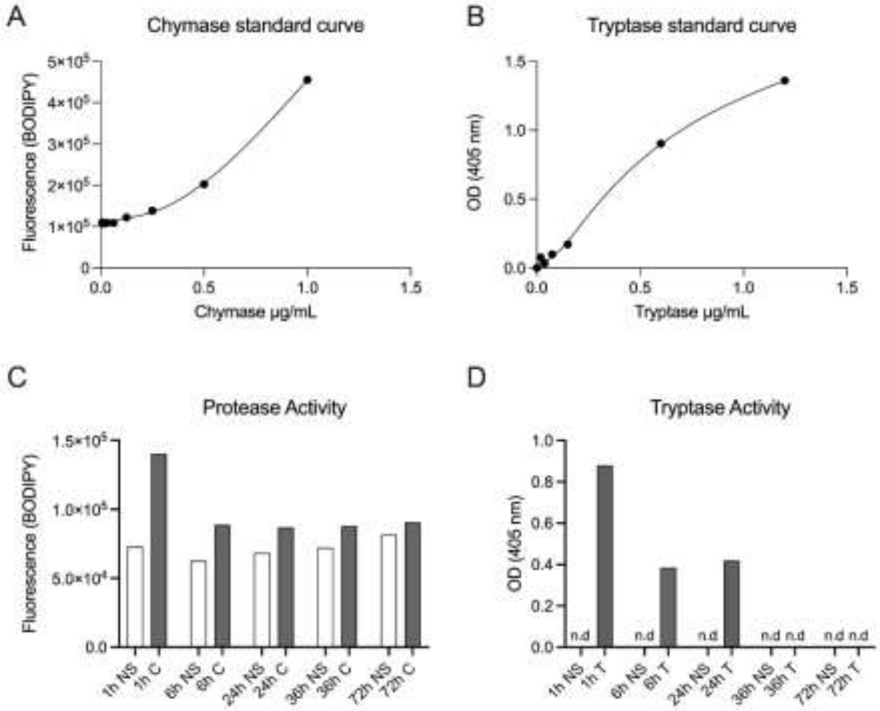

Figure S1. General protease activity and specific tryptase activity measured at 1, 6, 24, 36 and 72 hours after stimulation in cell supernatants. Standard curve for chymase (**A**) and tryptase (**B**). General protease activity in samples stimulated with chymase and non-stimulated cells (**C**). Tryptase activity in samples stimulated with tryptase and non-stimulated cells (**C**). n.d.: not detetctable.

1.2. Mast cell tryptase and chymase alters gene expression of motility associated genes in bronchial epithelial cells

An RT[2] profiler PCR array (PAHS-128Z) for motility associated genes was analyzed in BEAS2B after stimulation with tryptase, chymase or in combination. Tryptase significantly upregulated urokinase plasminogen activator surface receptor (PLAUR) (p=0.05) and Ezrin (EZR) (p=0.03) while downregulated matrix metallopeptidase 9 (MMP9) (p=0.006), phospholipase D1 (PLD1) (p=0.03) and WASP-family verprolin homologous protein 1 (WASF1) (p=0.008). The combination of tryptase and chymase significantly upregulated RAS p21 protein activator 1 (RASA1) (p=0.02) and signal transducer and activator of transcription 3 (STAT3) (p=0.04). Although not reaching statistical significance, chymase stimulation showed a trend towards upregulating transforming protein RhoA (RHO).

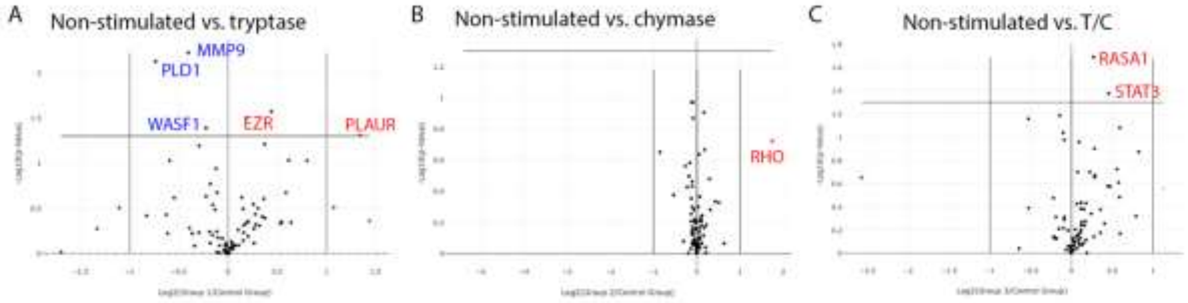

Figure S2. Tryptase and chymase induce alteration in motility associated genes in bronchial epithelial cells. Volcano plot of Log2 fold change (x-axis) and -Log10 significance (y-axis) for 84 motility associated genes in BEAS2B after stimulation with (**A**) tryptase, (**B**) chymase and (**C**) chymase and tryptase in combination (T/C). Fold change is represented in relation to non-stimulated cells. Horizontal line represents significant at p≤ 0.05. The two vertical lines represent a fold change of 2. Results are based on three independent experiments.
